# Disconnection Mechanism and Regional Cortical Atrophy Contribute to Impaired Processing of Facial Expressions and Theory of Mind in Multiple Sclerosis: A Structural MRI Study

**DOI:** 10.1371/journal.pone.0082422

**Published:** 2013-12-13

**Authors:** Andrea Mike, Erzsebet Strammer, Mihaly Aradi, Gergely Orsi, Gabor Perlaki, Andras Hajnal, Janos Sandor, Miklos Banati, Eniko Illes, Alexander Zaitsev, Robert Herold, Charles R. G. Guttmann, Zsolt Illes

**Affiliations:** 1 Division of Clinical and Experimental Neuroimmunology, Department of Neurology, University of Pecs, Pecs, Hungary; 2 Diagnostic Center of Pecs, University of Pecs, Pecs, Hungary; 3 Department of Psychiatry and Psychotherapy, University of Pecs, Pecs, Hungary; 4 Faculty of Public Health, University of Debrecen, Debrecen, Hungary; 5 Center for Neurological Imaging, Departments of Radiology and Neurology, Brigham and Women’s Hospital, Harvard Medical School, Boston, MA, United States of America; 6 MTA-PTE Clinical Neuroscience MR Research Group, Pecs, Hungary; 7 Department of Neurology, Institute of Clinical Research, Odense University Hospital, University of Southern Denmark, Odense, Denmark; Charité University Medicine Berlin, Germany

## Abstract

Successful socialization requires the ability of understanding of others’ mental states. This ability called as mentalization (Theory of Mind) may become deficient and contribute to everyday life difficulties in multiple sclerosis. We aimed to explore the impact of brain pathology on mentalization performance in multiple sclerosis. Mentalization performance of 49 patients with multiple sclerosis was compared to 24 age- and gender matched healthy controls. T1- and T2-weighted three-dimensional brain MRI images were acquired at 3Tesla from patients with multiple sclerosis and 18 gender- and age matched healthy controls. We assessed overall brain cortical thickness in patients with multiple sclerosis and the scanned healthy controls, and measured the total and regional T1 and T2 white matter lesion volumes in patients with multiple sclerosis. Performances in tests of recognition of mental states and emotions from facial expressions and eye gazes correlated with both total T1-lesion load and regional T1-lesion load of association fiber tracts interconnecting cortical regions related to visual and emotion processing (genu and splenium of corpus callosum, right inferior longitudinal fasciculus, right inferior fronto-occipital fasciculus, uncinate fasciculus). Both of these tests showed correlations with specific cortical areas involved in emotion recognition from facial expressions (right and left fusiform face area, frontal eye filed), processing of emotions (right entorhinal cortex) and socially relevant information (left temporal pole). Thus, both disconnection mechanism due to white matter lesions and cortical thinning of specific brain areas may result in cognitive deficit in multiple sclerosis affecting emotion and mental state processing from facial expressions and contributing to everyday and social life difficulties of these patients.

## Introduction

Cognitive decline is a common clinical manifestation of multiple sclerosis (MS) with an estimated prevalence between 40–65% [1], [2]. Previous studies investigating characteristics of cognitive dysfunction in MS found that working memory and information processing are most frequently impaired, followed by deficits in verbal and visuo-spatial learning and memory, executive functions, and visual processing [1], [2]. Quantitative MRI studies suggested relationship between cognitive deficit in MS and a wide range of pathologies: white matter (WM) lesion load, cortical lesion load, whole brain atrophy, cortical and deep gray matter atrophy, and diffuse abnormalities of brain tissue only revealed by techniques such as magnetization transfer or diffusion tensor MRI [3].

Deficits of general intellectual abilities significantly interfere with quality of life by impacting everyday life activities and decision-making [4], [5]. In addition, loss of employment status, restricted social activities, and difficulties in inter-personal relationships frequently occur during the disease [5].

Such functional limitations may also relate to deficits of social cognition. Social cognition is a human ability of making inferences about mental states of other people. An important aspect of social cognition is the capacity to interpret and predict mental states of other people in terms of thoughts, intentions, desires, and beliefs known as theory of mind (ToM), also referred as mentalizing. ToM is enabled by decoding nonverbal cues, such as facial expression, eye gaze, body gestures; and complex abstract reasoning about verbal information [6]. Social cognition might be independent or dissociable from general intelligence [7]. So far a few studies have investigated social cognitive abilities of patients with MS. These studies have demonstrated deficits both in facial emotion recognition and cognitive inferences about complex mental states of others [8], [9], [10], [11], [12], [13], [14], [15], [16].

The neural basis of ToM abilities has been widely investigated using advanced neuroimaging methods in healthy subjects and in clinical conditions showing social cognitive impairment, particularly in high-functioning autism and in schizophrenia [17], [18], [19], [20], [21]. These studies support the hypothesis that integrated fronto-temporal and temporo-parietal circuits are involved in mentalizing. Main hubs of these networks were found in the posterior superior temporal sulcus, temporo-parietal junction, temporal pole, medial prefrontal cortex, anterior cingulate cortex, orbitofrontal cortex, and inferior parietal lobule, as well as in the amygdala [6], [17], [22]. In addition, premotor and parietal regions constituting the putative mirror neuron system have been implicated when social meaning is extracted from nonverbal cues [23].

Previously, two functional MRI studies have investigated the brain activation during recognition of emotional face expressions in MS [12], [24]. One study found decreased insular and ventrolateral prefrontal cortex activation in patients with MS showing impaired recognition performance compared to the unimpaired MS group [12]. The other study reported an increased activation in the posterior cingulate cortex and precuneus interpreted as a compensatory mechanism [24].

Here, using quantitative MRI methods in patients with MS we investigated (i) the impact of WM lesion load, and cortical atrophy on ToM performance; (ii) we identified the topography of these pathologies to explore neuroanatomic substrates, which if damaged, impact mentalization ability.

## Materials and Methods

### Ethics Statement

The Research Ethical Committee of the Clinical Centre of the University of Pecs approved the study, and written informed consent was obtained from all participants. All patients with MS included in the study had complete clinical and neurological exam, during which capacity and ability to give consent for participation in the study had been evaluated. All of the included patients had been established to be fully able to give informed consent, and there was no need to involve guardians due to compromised capacity to consent. Healthy control volunteers had been recruited from university students and staff of the Medical School of Pecs, University of Pecs, and during an initial short interview capacity to be fully able to give informed consent had been established. All healthy participants gave written informed consent.

### Participants

Forty-nine white patients with MS according to revised McDonald criteria [25] were recruited from the Department of Neurology, University of Pecs. Demographic and clinical characteristics are presented in **Table 1**. Patients were eligible for the study if the disease was clinically stable for at least 30 days, they had no history of alcohol or drug dependence, major psychiatric illness, neurological disease (other than MS), and gross visual impairment. Each patient had clinical exam, neuropsychiatric testing, and brain MRI on the same day. Physical disability was assessed by the Expanded Disability Status Scale (EDSS) [26]. At the time of the study 40 patients (82%) received immunomodulatory treatment (glatiramer acetate, beta-interferon).

**Table 1:** Demographic data, clinical characteristics, results of social cognitive testing and psychometric assessment obtained in patients with multiple sclerosis and group of healthy controls recruited for neuropsychological testing.

	MS^1^ (n = 49)	Controls1 (n = 24)
**Gender:** female/male (%)	31/18 (63.3/36.7)	13/11 (54.2/45.8)
**Age** (y): range (mean±StD)	20–61 (39.82±9.31)	24–51 (36.71±7.27)
**Disease course**: RR^2^/SP^3^ (%)	44/5 (89.9/10.1)	NA
**Disease duration** (y): range (mean±StD)	0.5–21 (9.49±6.19)	NA
**EDSS** 4 **:** range (mean±StD)	0–6 (2.43±1.71)	NA
**Faces test:** range (median)	8–20 (17)	16–20 (19)
**Eyes test:** range (mean±StD)	16–30 (22.47±3.37)	19–31 (25.67±3.05)
**Faux pas test:** range (median)	3–10 (8)	5–10 (8)
**STAI** 5 **:** ratio of anxious subjects (%)	19/49 (38.78)	1/24 (4.17)
**BDI** 6 **:** ratio of depressed subjects (%)	9/49 (18.37)	0/24 (0)

Cut-off values were 16 and 54 for BDI and STAI, respectively.

^1^ multiple sclerosis.

^2^ relapsing-remitting.

^3^ secondary progressive.

^4^ Expanded Disability Status Scale.

^5^ Spielberg Trait Anxiety Inventory.

^6^ Beck Depression Inventory.

Two cohorts of healthy controls were used. For neuropsychological testing, we recruited 24 gender- and age-matched healthy volunteers with no previous history of neurological dysfunction, and normal neurological examination (**Table 1**). After we found that thickness of particular cortical areas correlated with Eyes test performance (see below) in the MS group, we recruited another control group to measure their cortical thickness by MRI: comparing MS and this control group, we assessed atrophy of these cortical areas in MS. This second control group of 18 healthy volunteers was gender and age matched to the first control group, and its performance in the Eyes test was similar (**Table 1**, **Table 2**).

**Table 2:** Results of Eyes test performance and MRI measures in patients with multiple sclerosis and group of healthy controls recruited for MRI examination.

	MS^1^ (n = 49)	Controls2 (n = 18)
**Gender:** female/male (%)	31/18 (63.3/36.7)	12/6 (66.7/33.3)
**Age** (y): range (mean±StD)	20–61 (39.82±9.31)	27–56 (38.22±8.34)
**Eyes test:** range (mean±StD)	16–30 (22.47±3.37)	21–29 (23.81±2.43)
**Total T1-lesion volume** (mm^3^): range (median)	111.6–20000.4 (1524.0)	NA
**Total T2-lesion volume** (mm^3^): range (median)	1362.4–110976.0 (20272.2)	NA
**Left FFA** 2 (mm): range (mean±StD)	1.74–3.60 (2.40±0.38)	1.75–3.41 (2.43±0.42)
**Left TP** 3 (mm): range (mean±StD)	1.55–4.09 (2.75±0.55)	2.29–4.64 (3.11±0.68)
**Right FEF** 4 (mm): range (mean±StD)	1.52–3.11 (2.26±0.35)	1.67–2.86 (2.22±0.37)

Cortical thickness data (left FFA, left TP, right FEF) have been obtained by the measure of the average cortical thickness of cortical areas showing correlation with Eyes test performance in the group of patients with multiple sclerosis.

^1^ multiple sclerosis.

^2^ Fusifrom Face Area.

^3^ Temporal pole.

^4^ Frontal Eye Field.

### Social Cognitive Testing and Psychometric Assessment

All tests were administered and have been validated in Hungarian.

#### Tests of emotion recognition and mental state attribution abilities from facial expressions

Computerized version of the Faces test [27], and the Eyes test [28] were completed in a single session. The stimuli were presented on separate slides after each other.

*Faces test*: The Faces test consists of 20 photographs of the face of the same white actress expressing different mental states [27]. Subjects were instructed to choose from two terms (one correct and one incorrect) presented simultaneously below the photograph the one, which best describes the presented facial expression. Each correct choice scored one point.

*Eyes test*: The Eyes test consists of 36 photographs depicting only the eye region of the face of different white individuals expressing various mental states [28]. Participants were asked to choose from 4 mental state terms (one correct and three incorrect) presented simultaneously at each corner of the photograph the one best describes the displayed thought or feeling. Each correct choice scored one point. In a control trial, subjects had to judge the gender of each person on the photograph to control for possible impairments in face perception, which could interfere with facial expressions recognition.

#### Test of mental state attribution abilities from verbal information

Perspective taking and reasoning about mental and emotional states of others was evaluated with the *Faux pas test* [29]. In a faux pas situation somebody unwittingly makes an awkward comment not realizing that it might hurt another person. To detect a faux pas, the participant has to simultaneously understand the knowledge or beliefs of both characters in the situation and appreciate the emotional states associated with them. We applied 5 Faux pas tasks, and 5 control tasks presented in random order using the same design. A short verbal story containing a faux pas or a control situation was displayed on computer screen. After reading a story, participants were asked to answer two questions to evaluate proper interpretation of the read situation. See example in **Text S1.** Correct comprehension scored one point. A control task was used to assess basic story comprehension.

#### Psychometric assessment

Depression was assessed with the Beck Depression Inventory (DBI) a validated screening tool for depressive symptoms [30]. The BDI evaluates self-reported responses to a multi-choice questionnaire containing 21 items. Anxiety was determined with the 20-item Spielberger Trait Anxiety Inventory (STAI) [31]. The STAI consists of anxiety trait and anxiety state evaluation on a 4-point rating scale. Cut-off values were 16 and 54 for BDI and STAI, respectively.

### MRI

#### MRI acquisition

MRI was performed on a 3T Magnetom TIM Trio whole-body MRI scanner (Siemens AG, Erlangen, Germany) with a 12-channel phased array head coil. The following sequences were performed: (1) T1-weighted three-dimensional (3D) Magnetization Prepared Rapid Gradient Echo (MPRAGE) (TR/TI/TE = 2300/900/1.9 ms, Flip Angle = 9°, 160 sagittal slices, slice thickness = 1.2 mm, field of view = 240×256 mm^2^, matrix size = 240×256, bandwidth = 240 Hz/pixel, scanning time ∼5 minutes); (2) single-slab 3D fast spin-echo Fluid-attenuated inversion recovery (FLAIR) (TR/TE/TI = 6000/2200/334 ms, 144 sagittal slices, slice thickness = 1.3 mm, field of view = 208×256 mm^2^, matrix size = 209×256, bandwidth = 780 Hz/pixel, scanning time ∼13 minutes).

#### MRI analysis

**Figure 1:**
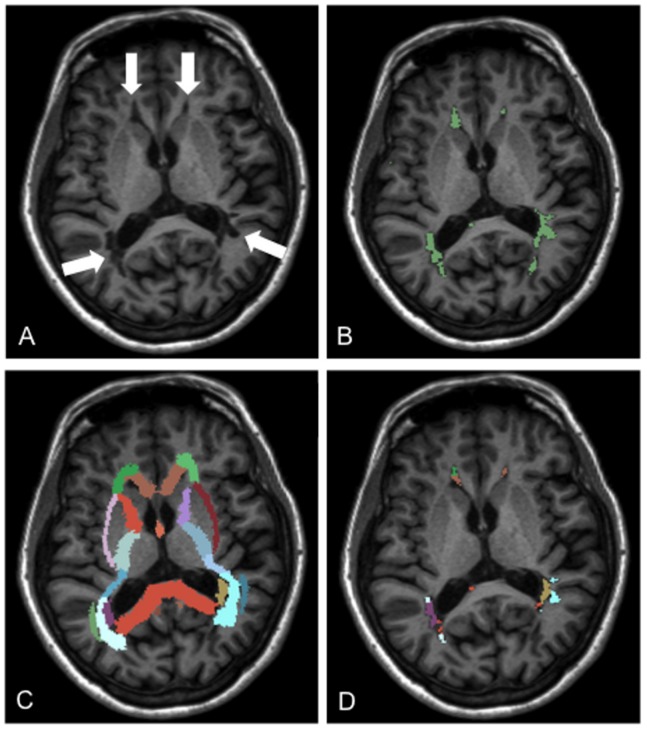
Assessment of total and regional T1 lesion volume in patients with multiple sclerosis. **A.** T1 hypointense lesions are shown on a representative grayscale MPRAGE image (white arrows). **B.** Binary lesion map generated from outlined T1 hypointense lesions visualized in green. **C.** WM fiber tract atlas registered into native space of the MPRAGE image. **D.** Labeled lesion map derived from the binary lesion map after parcellation of the lesions with the use of the WM fiber tract atlas. The labeled lesion map was used to measure regional T1 white matter lesion volumes within each individual fiber bundle.

*Total lesion load measurement*: Field inhomogeneity correction was performed for FLAIR images by applying an automated algorithm based upon entropy minimization [32]. A neurologist [AM] with clinical and imaging experience in MS blinded to clinical and neuropsychological findings outlined the lesions, and generated binary lesion maps from MPRAGE and FLAIR images, separately using a semi-automated thresholding technique with the software 3D-Slicer, version 3.4 (http://www.slicer.org). The method relies on user-guided specification of the intensity threshold range, which is manually adjustable during lesion segmentation. The algorithm labels all pixels with signal intensity within the set threshold range in the lesion area identified manually by the user (**Figure 1**).

*Analysis of regional white matter lesions within the white matter fiber bundles*: MRI images were processed with the freely available Software Library tools of FMRIB (FSL, http://www.fmrib.ox.ac.uk/fsl). For parcellation of the WM lesions the International Consortium for Brain Mapping (ICBM) DTI-81 Atlas [33] was overlaid onto the binary lesion maps generated from MPRAGE and FLAIR images of each patients. This WM fiber tract atlas, which was originally derived from a diffusion tensor imaging database of 81 normal subjects and normalized to the MNI-ICBM template parcellates the WM of the two hemispheres into 50 fiber bundles (**Figure 1**). During MRI processing brain extraction was performed on patients’ MPRAGE and FLAIR images using Brain Extraction Tool [34]. MPRAGE images were then spatially registered into ICBM standard space (MNI152_T1_1 mm_brain, 1×1×1 mm^3^/voxel) using a two-step process. First, brain-extracted MPRAGE images were registered to the MNI152 standard brain image (12 degrees-of-freedom linear fit, correlation ratio cost function) using the FMRIB Linear Image Registration Tool (FLIRT) [35]. Second, the registration was further refined by nonlinear registration using FMRIB’s Non-linear Image Registration Tool. Subsequently, an inverse transformation was applied to warp the ICBM DTI-81 Atlas labels into the native space of MPRAGE images. The brain-extracted FLAIR images were registered to brain-extracted MPRAGE images (6 degrees-of-freedom linear fit, correlation ratio cost function). To warp the ICBM DTI-81 Atlas labels to native space of FLAIR images the nonlinear transformation from standard space to native MPRAGE image space was concatenated with the inverse of linear transformation from native FLAIR space to native MPRAGE space. The quality of all registrations was visually evaluated. The T1 and T2 binary lesion maps were labeled in their own native space using the transformed ICBM DTI-81 Atlas. The labeled lesion maps were used to measure regional T1-weighted WM lesion volume (rT1LV) and regional T2-weighted WM lesion volume (rT2LV) in the individual fiber bundles (**Figure 1**). We assessed the volume of each individual fiber bundle to calculate their relative lesion content (**Table S1, Table S2**). All volume measures were performed in patients’ native space.

*Cortical thickness measures*: We measured the thickness of the neocortex throughout the whole brain on 3D MPRAGE images using the automated reconstruction algorithm of the software FreeSurfer, version 4.5 (http://surfer.nmr.mgh.harvard.edu) [36], [37], [38]. The entire cortex of the subjects was visually inspected and in the MS group errors due to misclassifications of WM lesions located close to the cortex were manually edited and automatically corrected. In the MS group, the relationship between performances in each mentalization tests and cortical thickness was computed at each vertex point using general linear models corrected for age [39]. Cortical regions showing correlation at p<0.001 were displayed as significance maps on the FreeSurfer averaged brain surface. Regions covering surface area ≥25 mm^2^ were manually outlined (region of interest [ROI]), and automatically mapped back on the brain surface of each patient with MS and participant of the healthy control group recruited for MRI examination. The average thickness of ROIs was calculated for each participant.

### Statistical Analysis

Statistical analyses were performed using SPSS software version 18.0 (SPSS, Chicago, IL, USA). Distribution of the standardized residuals of the dependent variables resulting from the applied regression models of interest were analyzed by the Shapiro-Wilk test. In cases of non-normal distribution, square root and logarithmic transformations of the dependent variable data were performed, and distributions of the standardized residuals were examined with the same statistical method. Dependent variables showing non-normal distribution were dichotomized by the median.

Mentalization performance of patients with MS and healthy controls were compared using ANOVA for Eyes test, and binary logistic regression for Faces test and Faux pas test accounting for confounding factors (gender, anxiety, depression).

Relationships between mentalization performance of patients with MS and total T1- and T2-weighted WM lesion volumes (tT1LV, tT2LV, respectively), regional T1- and T2LVs (rT1LV, rT2LV, respectively), and cortical thickness of the ROIs accounting for confounding factors of gender, EDSS, anxiety, and depression were analyzed with ANCOVA. Correlations between Faux pas test and tT1- and tT2LV accounting for the confounding factors were analyzed by binary logistic regression. Multiple comparisons were corrected with Bonferroni method.

Cortical thicknesses of the ROIs correlating with the Eyes test were compared between control group recruited for MRI examination and total MS cohort, and MS subgroups with and without social cognitive impairment using ANCOVA accounting for age. We defined impaired social cognitive performance among patients with MS as test performance 1 standard deviation below the mean of the MRI control group’s performance.

To assess the contribution of regional WM lesion volumes and cortical thicknesses of ROIs to the variance of Eyes test performance, ANCOVA analyses were performed corrected for gender, EDSS, anxiety, and depression.

All statistical testing were performed at Type I error = 0.05 for single tests or family-wise Type I error = 0.05 for multiple testing.

## Results

### Comparison of Social Cognitive Performance between Patients with MS and Healthy Controls

Results of social cognitive testing and psychometric assessment are presented in **Table 1**. After correction for anxiety, depression, and gender, patients with MS performed significantly poorer in the Faces test (p = 0.006, OR = 0.144) and in the Eyes test (p = 0.007, R^2^ = 0.276), but not in Faux pas test compared to the group of 24 healthy controls.

### Impact of White Matter Lesion Volumes on Mentalization Performance of Patients with MS

We examined MRI correlations with all three mentalization tests (Eyes test, Faces test, Faux pas test) even though performance in Faux pas test was not deficient.

#### Effect of total white matter lesion volumes

Descriptive data of the total lesion load measures are presented in **Table 2**. After correction for the confounding factors (gender, EDSS, anxiety, and depression), Faces test and Eyes test performance showed correlation with tT1LV (p = 0.003, R^2^ = 0.315, p = 0.034, R^2^ = 0.230, respectively), but not with tT2LV. No relationship was found between Faux pas test performance and any of the two total WM lesion measures.

#### Effect of regional white matter lesion volumes of major inter-connecting tracts

We investigated the impact of regional T1-, and T2-lesions located within the inter-connecting fiber bundles on mentalization performance of patients with MS. Only tracts containing lesions in a considerable number of patients (≥25%, ≥12 patients) were included in the statistical analysis: for T1-lesions 21, and for T2-lesions 37 out of 50 fiber bundles fulfilled these criteria (**Table S1, Table S2**). After correction for confounding variables and using Bonferroni adjustment (for rT1LVs: p<0.0024, for rT2LVs: p<0.0014) the Faces test performance showed significant linear negative correlation with rT1LV of the genu of corpus callosum (GCC) (p<0.001, R^2^ = 0.415), splenium of corpus callosum (SCC) (p<0.002, R^2^ = 0.332), left anterior corona radiata (ACR) (p<0.001, R^2^ = 0.41), right sagittal stratum including the inferior longitudinal fasciculus (ILF) and inferior fronto-occipital fasciculus (IFOF) (p<0.001, R^2^ = 0.407), and left and right uncinate fasciculus (UF) (p<0.002, R^2^ = 0.325, p<0.001, R^2^ = 0.468, respectively) (**Table S1**). Faces test performance also showed correlation with rT2LV of the GCC (p<0.001, R^2^ = 0.369) and left Fornix (cres)/Stria terminalis (p<0.001, R^2^ = 0.471) (**Table S2**). The Eyes test performance correlated with rT1LV of the splenium of corpus callosum (SCC) (p<0.002, R^2^ = 0.317) (**Table S1**), and did not show correlation with rT2LV of any fiber tract (**Table S2**). The Faux pas test performance did not correlate with rT1LV or rT2LV of any fiber tract (**Table S1, Table S2**).

### Impact of Cortical Thickness on Mentalization Performance of Patients with MS

**Figure 2:**
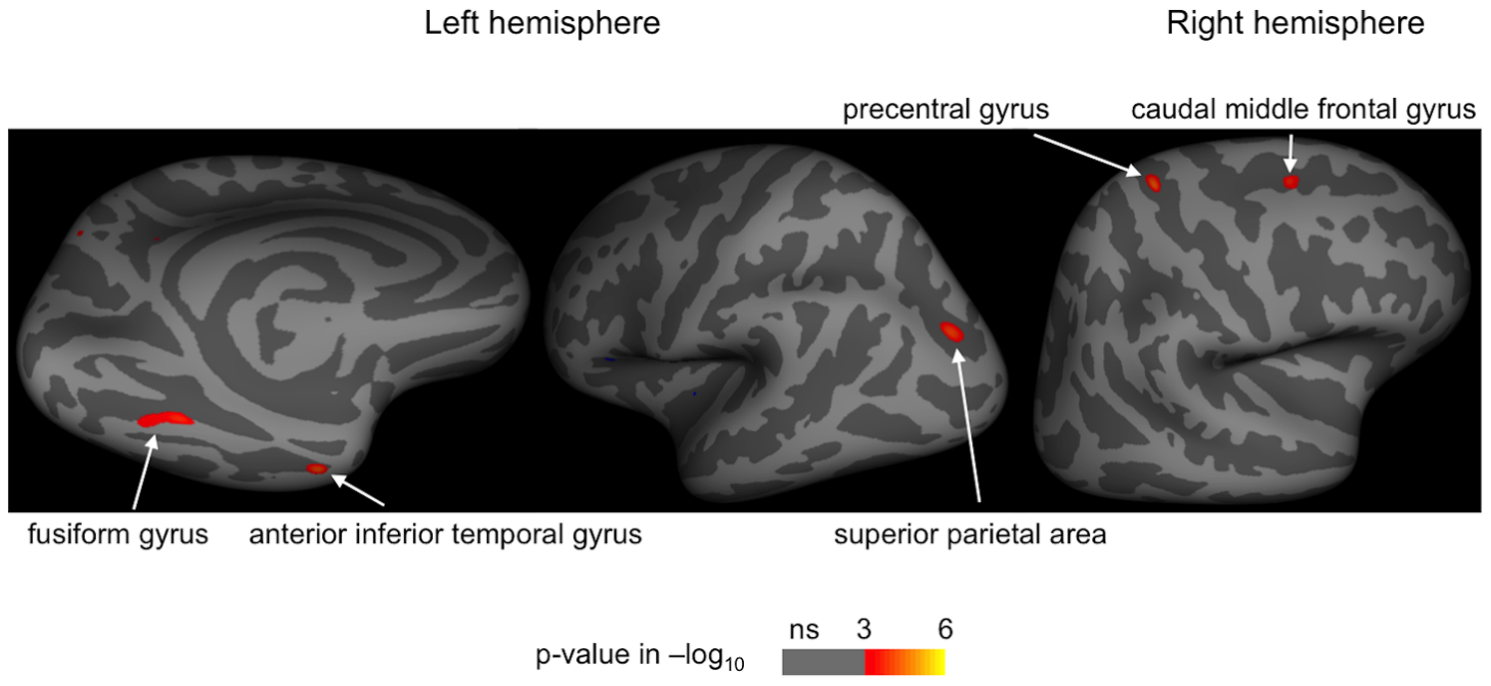
Cortical areas of which thickness correlated with Eyes test performance in patients with multiple sclerosis. Significance map from general linear model analysis of 49 patients with multiple sclerosis is displayed at each vertex of the inflated standardized brain. After correction for the effect of age, the Eyes test performance showed significant correlations (p<0.001) with cortical thickness of areas shown on the figure. P-values are presented in the color bar (logarithmic value).

We explored the entire cortex to find correlations between cortical thinning and mentalization performance after controlling for age. Poor Faces test performance correlated with thinning of 3 cortical areas: left and right fusiform face area (FFA) of the fusiform gyrus (BA19), and an area in the right entorhinal cortex (BA28). After correction for anxiety, depression, gender, and EDSS and using a Bonferroni adjustment (p<0.017), all ROIs invariably remained significant (**Table 3**). Poor Eyes test performance showed association with thinning of 5 cortical areas (**Figure 2** **,** **Table 3** **)**. After correction for the confounding factors and using a Bonferroni adjustment (p<0.01), ROIs at the left temporal pole in the anterior inferior temporal gyrus (Brodmann area [BA] 20), left FFA of the fusiform gyrus (BA19), and right caudal middle frontal gyrus (frontal eye field [FEF], BA8) remained significant (**Table 3**). Faux pas tests did not correlate significantly with cortical thickness of any brain areas.

**Table 3:** Data of cortical thickness and area of brain regions showing correlation with the performance in the Faces test and Eyes test in patients with multiple sclerosis accounting for age.

Mentalizationtest	Brain region	Thickness (mm): range(mean±StD)	Area (mm^2^) range(mean±StD)	ANCOVA (Bonferronicorrected p<0.01)	
				p	R^2^
**Faces test**	Left fusiform gyrus (fusiform face area)	1.834–2.881 (2.453±0.25)	44–106 (72.73±16.76)	**0.001**	0.345
	Right fusiform gyrus (fusiform face area)	0.370–2.897 (2.19±0.37)	63–130 (88.16±14.84)	**0.004**	0.304
	Right entorhinal cortex	1.815–4.031 (3.262±0.36)	199–390 (291.67±33.68)	**<0.001**	0.460
**Eyes test**	Left superior parietal area	1.156–2.529 (1.903±0.33)	36–72 (50.67±9.10)	0.050	0.217
	Left fusiform gyrus (fusiform face area)	1.737–3.596 (2.397±0.38)	20–68 (35.84±9.84)	**0.007**	0.282
	Left anterior inferior temporal gyrus(temporal pole)	1.552–4.088 (2.748±0.55)	32–106 (58.82±18.24)	**<0.001**	0.380
	Right caudal middle frontal gyrus (frontaleye field)	1.523–3.112 (2.255±0.35)	20–45 (31.27±6.42)	**0.008**	0.278
	Right precentral gyrus	1.110–1.933 (1.434±0.16)	37–75 (49.88±6.35)	0.020	0.247

The relationships between average cortical thickness of the brain regions and mentalization test performances were further analyzed to account for confounding factors (gender, EDSS, anxiety, and depression) potentially impacting mentalization, and were corrected for multiple comparisons using Bonferroni method (p<0.017 for the Faces test and p<0.01 for Eyes test). Significant correlations are shown in bold format.

### Comparison of Cortical Thickness between Patients with MS and Healthy Controls

To assess whether cortical ROI regions showing correlation with poor performance in the Eyes test in the MS group are atrophic, we measured their thickness in the control group of 18 healthy subjects recruited for MRI exam. When controlling for gender, ANOVA test revealed significantly better Eyes test performance in this control group compared to the MS group (p = 0.044, R^2^ = 0.082) (**Table 2**). Cortical thickness data of the control group are shown in **Table 2**. The ROI in the left anterior inferior temporal gyrus was significantly thinner in the MS group, and the ROI in the left FFA was significantly thinner in the MS subgroup with impaired Eyes test performance (19 out of 49 patients) compared to the healthy control group. The thickness of the right FEF ROI did not differ significantly (**Table 4**).

**Table 4:** Comparison of mean cortical thickness of brain areas showing correlation with Eyes test performance between patients with multiple sclerosis and healthy control group.

Region of interest	Talairach coordinates			ANCOVA^a^ p (R^2^)		
	x	y	z	MS^1^ vs. HC^2^	Impaired MS vs. HC	Non-impaired MS vs. HC
left fusiform face area (BA19)	−30	−53	−13	0.755 (0.003)	**0.043** (0.125)	0.552 (0.017)
left temporal pole (BA20)	−41	−9	−36	**0.038** (0.101)	**0.026** (0.182)	0.164 (0.055)
right frontal eye field (BA8)	28	11	45	0.762 (0.002)	0.223 (0.056)	0.285 (0.028)

^a^ comparisons between patients with MS and HC were controlled for age.

^1^ multiple sclerosis.

^2^ healthy controls.

According to the performance in the Eyes test, patients with multiple sclerosis (n = 49) were divided into impaired (n = 19) and non-impaired (n = 30) subgroups. Impaired social cognitive performance was defined as test performance 1 standard deviation below the mean of the performance of the healthy control group (n = 18) recruited for the cortical thickness measure.

### Contribution of MRI Variables to the Variance of Mentalization Performance

Multivariate regression analysis of the contribution of rT1LVs and cortical ROIs showing significant correlation with the Faces test performance showed that the rT1LV of the left UF independently predicted the Faces performance (p = 0.031) and accounted for a 65.1% variance of the performance. Regional T1LV of the SCC (p = 0.046), cortical thickness of ROIs in left FFA (p = 0.024), and left temporal pole (p = 0.002) independently predicted the Eyes test performance, and accounted for 59.1% of the variance of the performance. The right premotor area did not remain significant (p = 0.088).

## Discussion

The present MRI study investigated the impact of WM lesion burden and cortical thinning on mentalization ability of patients with MS. Exploration of brain areas, which structural damage associate with impaired mentalization performance in MS may point to neurophysiologic mechanisms involved in processing of information of social relevance. In contrast to autism [19], MS provided a model to investigate the effect of structural tissue damage on mentalization in subjects with supposedly normal brain development.

Mentalization ability was evaluated by tests of emotion recognition from facial expressions (Faces test), mental state decoding from eye gazes (Eyes test), and reasoning about mental states from verbal stories (Faux pas test). After correction for confounding effects of anxiety, depression, and gender, patients with MS performed significantly poorer in the Faces test and Eyes test compared to healthy subjects. These results obtained with an MS cohort different from our previous study confirmed those findings [9], and are in line with other previous reports [8], [10], [11], [12], [13], [14], [15], [16].

Poorer emotion and mental state recognition performances from facial expressions and eye gazes showed relationship with both WM and cortical gray matter damages measured by MRI in patients with MS. The multivariate regression analysis has demonstrated that both regional T1LV and cortical thinning of certain focal brain areas may independently compromise the mentalization performance, and as such both a disconnection mechanism due to WM damage and the cortical gray matter damage may contribute to impaired cognitive performance in MS.

After correction for confounding effects of gender, EDSS, anxiety, and depression, both poorer Faces test and Eyes test performance showed correlation with higher tT1LV. To examine whether these relationships are specifically associated with damages of specific WM fiber tracts, we measured the WM lesion volumes in each major fiber bundle anatomically identified by a WM fiber tract atlas [33] (**Figure 1**). To our knowledge, this method has not been used before to correlate neuropsychological data with regional lesion load in patients with MS. We have found that poorer Faces test and Eyes test performances were related to rT1LV of association fiber tracts interconnecting cortical regions related to visual and emotion processing (GCC, SCC, right ILF and IFOF, and right and left UF). The SCC interconnects identical posterior cortical areas (occipital, parietal, and temporal lobes) of the two hemispheres largely involved in visual perception [40]. Damage in the SCC showing association with both the Faces and the Eyes test performance may disrupt the interhemispheric integration of visual information resulting in deficient emotion recognition and mental state decoding from facial expressions [41], [42], [43]. This finding is concordant with previous studies demonstrating that higher-order visual functions are impaired in patients with complete callosotomy [44], and individuals with agenesia of the corpus callosum perform poorer than controls in facial emotion recognition tests [45]. Poorer Faces test performance was also related to higher T1-lesion load of the right IFL and IFOF. The ILF projects from the occipital cortex to the anterior temporal lobe and amygdala. The IFOF connects the occipital cortex to the orbito-frontal cortex passing through the medial temporal cortex [46], [47]. One study has provided evidence that damages of these pathways are associated with impaired emotion recognition from facial expressions [47]. Both rT1- and rT2LV of the GCC correlated with the Faces test performance of patients with MS. The GCC interconnects identical prefrontal and premotor cortical areas [40], and as such contributes to integrative processes of behavioral, emotional, and cognitive functions. The UF projects between anterior temporal cortex and prefrontal cortex with prominent projections to orbito-frontal areas [48], and is known to be involved in the emotion processing. Regional T1LV of left ACR also showed correlation with Faces test performance. The ACR projects between frontal cortex and subcortical gray matter structures [49], and was found to be part of the executive attention network, play an important role in the mediation of processing speed and decision-making. All of these cognitive functions may be required for proper solving of the Faces test [49], [50].

With the exceptions of the relationships between Faces test performance and rT2LV of the GCC and fornix/stria terminalis, nor total and regional T2LV showed correlations with performances of emotion and mental state recognition from facial expressions. This may presumably be explained by the finding that T1-hypointense lesions reflect more directly the severity of myelin and/or axonal loss [51]. Previously, T1-hypointense lesions have been found as better predictors of cognitive impairment in MS [51], [52].

Cortical gray matter thinning was also found to relate to mentalization performance of patients with MS. Secondary neurodegeneration resulted from axonal injury in WM lesions, cortical lesion formation, and primary neurodegenerative processes are presumed to play a role in cortical atrophy developing in MS [53], [54]. Correlation between neocortical volume loss and cognitive decline in MS has been previously demonstrated [3], [53]. Cortical thickness related to the Eyes test performance was found atrophic at the left temporal pole in the MS group when comparing to healthy controls and in the left FFA in the subgroup of patients with MS with significantly impaired Eyes test performance (**Table 4**).

All cortical areas identified in our study have been implicated as neural nodes of large-scale neural networks involved in facial expression and emotion processing, and in social cognition [17], [22], [41], [43], [55]. Both poor Faces test and Eyes test performances correlated with cortical thinning of the FFA (**Table 3**). The FFA is considered as the core area of face perception, and its involvement in facial emotion recognition has been demonstrated as well [41], [43], [55], [56]. Faces test performance was also related to cortical thickness of the right entorhinal cortex (**Table 3**) implicated in the emotion processing. The Eyes test performance showed correlation with thickness of the left anterior inferior temporal gyrus at the temporal pole (**Table 3**). The temporal pole is a known area of mentalization by confronting the actually perceived social and emotional signals with stored general knowledge about the world including autobiographical memory and social scripts [17]. For efficient mental state attribution in the Eyes test the visual stimuli are explicitly matched with verbally described mental states, and such integration of visual and contextual information might be enabled by the temporal pole. This finding is supported by a recent study showing task-related connectivity between the temporal pole and fusiform gyrus during motivated processing of emotional faces [57].

Correlation between cortical thickness of the right premotor FEF (BA8) and Eye test performance (**Table 3**) may be linked to the somatotopic organized mirror neuron system. The putative human mirror neuron system involves premotor and parietal regions [19], [23], [58], and is presumably involved in the processing of socially relevant nonverbal information [23], [59], [60]. Recent studies emphasize mirror neuron mechanism in the premotor cortex when mental states are decoded from facial expressions [57], [58], [59], [61]. Premotor mirror neurons fire with an identical pattern what would be required to the motor execution of an observed facial expression [19], [58]. The internally simulated facial expression is matched with a corresponding affective state experienced previously by the observer, and this enables the understanding of the observed mental state [58]. In the Eyes test participants have to decode social contents from eye gazes. We presume that cortical thinning in the FEF results in an impaired simulation mechanism of the somatotopic-organized mirror neurons firing when eye gazes are observed. This result is in line with a report demonstrating somatotopically organized activation of premotor and parietal areas when viewing mouth, hand, and foot motor actions [62].

Emotion decoding and mental state attribution from facial expressions are processed by large-scale neuronal networks specialized for information with social relevance [42], [43]. Nevertheless, functioning of these pathways is dependent on the primary visual perception. Recent studies in MS and other conditions with brain damage provided evidence that emotion recognition from faces may be impaired even with intact face identification ability [12], [13], [16], [47] suggesting specialized neural processing of facial expressions mediating emotions and mental states. Damages in the SCC, ILF, IFOF, and FFA may cause deficits in higher-order visual and emotion processing. In addition, damage in the FEF and temporal pole may cause deficit in brain functions specialized for mentalization.

Recent studies have shown relationship between facial expression recognition deficit and other cognitive domains characteristically impaired in MS [8], [10], [11], [16]. However, in other studies mentalization deficit in MS was independent from decline in other general cognitive domains [13], [14]. Multiple localizations of lesions in MS may affect different neural networks at the same time. Impairments in face processing, mental state attribution system, and general cognitive functions (visual information processing speed, attention, executive function) may additively contribute to deficits of mental state decoding from visual clues in MS.

Except of the temporal pole, we did not find correlations between mentalization performance and brain areas, which have been previously implicated in neural circuits for making inferences about mental states. This may be due to task-specific reasons: the Eyes test and Faces test does not need the processing of contextual social cues, which would engage these centers.

Faux pas performance was not different between patients with MS and healthy controls. Sensitivity of the Faux pas test to detect verbal mentalization deficit in MS has been found low [11], [14]. In addition, mentalization ability of patients with MS may be more vulnerable in tasks, which require visual information processing.

As a limitation of our study the healthy control group recruited for MRI scanning was not tested for anxiety and depression. Therefore we could not include mood state indicators as confounding factors into the statistical analysis and the model can only explain 8.2% of the variation of the Eyes test performance. Finally, we are aware that further studies with higher number of participants are needed to reproduce the findings and corroborate the results.

## Conclusion

Our results demonstrated impaired emotion and mental state recognition ability from facial expressions in patients with MS. Cortical thickness measure throughout of the whole brain on a vertex-to-vertex basis and lesion content assessment of each major fiber bundle in patients with MS without *a priori* hypothesis about possible anatomic substrates relating to mentalization performance have provided evidence for the significance of specific WM tracts and cortical brain areas associating with emotion and mental state decoding from facial expressions and mentalization function. Disconnection mechanism related to WM lesions and gray matter damage in cortical hubs constituting neural networks may independently compromise cognitive function in MS.

## Supporting Information

Table S1 **Regional T1-lesion content of the white matter tracts from the ICBM DTI-81 Atlas, and correlations between mentalization test performance and regional T1-lesion loads in patients with multiple sclerosis.** The volumes of each individual fiber bundle were assessed, and then their relative lesion content was calculated. Correlation between regional T1-lesion volumes and the social cognition test performances were analyzed with ANCOVA controlling for gender, EDSS, depression, and anxiety. P-value after Bonferroni correction was p<0.0024. Significant correlations are indicated with bold format.
(DOCX)

Table S2 **Regional T2-lesion content of the white matter tracts from the ICBM DTI-81 Atlas, and correlations between mentalization test performance and regional T2-lesion loads in patients with multiple sclerosis.** The volumes of each individual fiber bundle were assessed, and then their relative lesion content was calculated. Correlation between regional T2-lesion volumes and social cognition test performances were analyzed with ANCOVA controlling for gender, EDSS, depression, and anxiety. P-value after Bonferroni correction was p<0.0014. Significant correlations are indicated with bold format.
(DOCX)

Text S1 **Example from the Faux pas test.** The presented story contains a faux pas situation.
(DOCX)

## References

[1] Rao SM, Leo GJ, Bernardin L, Unverzagt F (1991) Cognitive dysfunction in multiple sclerosis. I. Frequency, patterns, and prediction. Neurology 41: 685–691. 2027484 10.1212/wnl.41.5.685

[2] Benedict RH, Cookfair D, Gavett R, Gunther M, Munschauer F, et al (2006) Validity of the minimal assessment of cognitive function in multiple sclerosis (MACFIMS). J Int Neuropsychol Soc 12: 549–558. 16981607 10.1017/s1355617706060723

[3] Filippi M, Rocca MA, Benedict RH, DeLuca J, Geurts JJ, et al (2010) The contribution of MRI in assessing cognitive impairment in multiple sclerosis. Neurology 75: 2121–2128. 21135387 10.1212/WNL.0b013e318200d768 PMC3385423

[4] Chiaravalloti ND, DeLuca J (2008) Cognitive impairment in multiple sclerosis. Lancet Neurol 7: 1139–1151. 19007738 10.1016/S1474-4422(08)70259-X

[5] Langdon DW (2011) Cognition in multiple sclerosis. Curr Opin Neurol 24: 244–249. 21519256 10.1097/WCO.0b013e328346a43b

[6] Stone VE, Baron-Cohen S, Knight RT (1998) Frontal lobe contributions to theory of mind. J Cogn Neurosci 10: 640–656. 9802997 10.1162/089892998562942

[7] Shamay-Tsoory SG, Tomer R, Aharon-Peretz J (2005) The neuroanatomical basis of understanding sarcasm and its relationship to social cognition. Neuropsychology 19: 288–300. 15910115 10.1037/0894-4105.19.3.288

[8] Henry JD, Phillips LH, Beatty WW, McDonald S, Longley WA, et al (2009) Evidence for deficits in facial affect recognition and theory of mind in multiple sclerosis. J Int Neuropsychol Soc 15: 277–285. 19203428 10.1017/S1355617709090195

[9] Banati M, Sandor J, Mike A, Illes E, Bors L, et al (2010) Social cognition and Theory of Mind in patients with relapsing-remitting multiple sclerosis. Eur J Neurol 17: 426–433. 19922457 10.1111/j.1468-1331.2009.02836.x

[10] Jehna M, Neuper C, Petrovic K, Wallner-Blazek M, Schmidt R, et al (2010) An exploratory study on emotion recognition in patients with a clinically isolated syndrome and multiple sclerosis. Clin Neurol Neurosurg 112: 482–484. 20399006 10.1016/j.clineuro.2010.03.020

[11] Ouellet J, Scherzer PB, Rouleau I, Metras P, Bertrand-Gauvin C, et al (2010) Assessment of social cognition in patients with multiple sclerosis. J Int Neuropsychol Soc 16: 287–296. 20167136 10.1017/S1355617709991329

[12] Krause M, Wendt J, Dressel A, Berneiser J, Kessler C, et al (2009) Prefrontal function associated with impaired emotion recognition in patients with multiple sclerosis. Behav Brain Res 205: 280–285. 19686782 10.1016/j.bbr.2009.08.009

[13] Phillips LH, Henry JD, Scott C, Summers F, Whyte M, et al (2011) Specific impairments of emotion perception in multiple sclerosis. Neuropsychology 25: 131–136. 21090898 10.1037/a0020752

[14] Henry A, Tourbah A, Chaunu MP, Rumbach L, Montreuil M, et al (2011) Social cognition impairments in relapsing-remitting multiple sclerosis. J Int Neuropsychol Soc 17: 1122–1131. 22014035 10.1017/S1355617711001147

[15] Beatty WW, Goodkin DE, Weir WS, Staton RD, Monson N, et al (1989) Affective judgments by patients with Parkinson’s disease or chronic progressive multiple sclerosis. Bulletin of the Psychonomic Society 27: 361–364.

[16] Prochnow D, Donell J, Schafer R, Jorgens S, Hartung HP, et al (2011) Alexithymia and impaired facial affect recognition in multiple sclerosis. J Neurol 258: 1683–1688. 21442462 10.1007/s00415-011-6002-4

[17] Frith CD, Frith U (2006) The neural basis of mentalizing. Neuron 50: 531–534. 16701204 10.1016/j.neuron.2006.05.001

[18] Schulte-Ruther M, Greimel E, Markowitsch HJ, Kamp-Becker I, Remschmidt H, et al (2011) Dysfunctions in brain networks supporting empathy: an fMRI study in adults with autism spectrum disorders. Soc Neurosci 6: 1–21. 20945256 10.1080/17470911003708032 PMC3046624

[19] Williams JH (2008) Self-other relations in social development and autism: multiple roles for mirror neurons and other brain bases. Autism Res 1: 73–90. 19360654 10.1002/aur.15

[20] Kuperberg GR, Broome MR, McGuire PK, David AS, Eddy M, et al (2003) Regionally localized thinning of the cerebral cortex in schizophrenia. Arch Gen Psychiatry 60: 878–888. 12963669 10.1001/archpsyc.60.9.878

[21] Herold R, Feldmann A, Simon M, Tenyi T, Kover F, et al (2009) Regional gray matter reduction and theory of mind deficit in the early phase of schizophrenia: a voxel-based morphometric study. Acta Psychiatr Scand 119: 199–208. 19016669 10.1111/j.1600-0447.2008.01297.x

[22] Adolphs R, Baron-Cohen S, Tranel D (2002) Impaired recognition of social emotions following amygdala damage. J Cogn Neurosci 14: 1264–1274. 12495531 10.1162/089892902760807258

[23] Rizzolatti G, Craighero L (2004) The mirror-neuron system. Annu Rev Neurosci 27: 169–192. 15217330 10.1146/annurev.neuro.27.070203.144230

[24] Jehna M, Langkammer C, Wallner-Blazek M, Neuper C, Loitfelder M, et al (2011) Cognitively preserved MS patients demonstrate functional differences in processing neutral and emotional faces. Brain Imaging Behav 5: 241–251. 21656213 10.1007/s11682-011-9128-1

[25] Polman CH, Reingold SC, Edan G, Filippi M, Hartung HP, et al (2005) Diagnostic criteria for multiple sclerosis: 2005 revisions to the “McDonald Criteria”. Ann Neurol 58: 840–846. 16283615 10.1002/ana.20703

[26] Kurtzke JF (1983) Rating neurologic impairment in multiple sclerosis: an expanded disability status scale (EDSS). Neurology 33: 1444–1452. 6685237 10.1212/wnl.33.11.1444

[27] Baron-Cohen S, Wheelwright S, Jolliffe T (1997) Is there a “language of the eyes”? Evidence from normal adults, and adults with autism or Asperger syndrome. Visual Cognition 4: 311–331.

[28] Baron-Cohen S, Wheelwright S, Hill J, Raste Y, Plumb I (2001) The “Reading the Mind in the Eyes” Test revised version: a study with normal adults, and adults with Asperger syndrome or high-functioning autism. J Child Psychol Psychiatry 42: 241–251. 11280420

[29] Baron-Cohen S, O’Riordan M, Stone V, Jones R, Plaisted K (1999) Recognition of faux pas by normally developing children and children with Asperger syndrome or high-functioning autism. J Autism Dev Disord 29: 407–418. 10587887 10.1023/a:1023035012436

[30] Beck AT, Ward CH, Mendelson M, Mock J, Erbaugh J (1961) An inventory for measuring depression. Arch Gen Psychiatry 4: 561–571. 13688369 10.1001/archpsyc.1961.01710120031004

[31] Spielberger CD, Gorsuch RL, Lushene PR, Vagg PR, Jacobs AG (1983) Manual for the Sate-Trait Anxiety Inventory: Consulting Psychologists Press.

[32] Sled JG, Zijdenbos AP, Evans AC (1998) A nonparametric method for automatic correction of intensity nonuniformity in MRI data. IEEE Trans Med Imaging 17: 87–97. 9617910 10.1109/42.668698

[33] Mori S, Oishi K, Jiang H, Jiang L, Li X, et al (2008) Stereotaxic white matter atlas based on diffusion tensor imaging in an ICBM template. Neuroimage 40: 570–582. 18255316 10.1016/j.neuroimage.2007.12.035 PMC2478641

[34] Smith SM (2002) Fast robust automated brain extraction. Hum Brain Mapp 17: 143–155. 12391568 10.1002/hbm.10062 PMC6871816

[35] Jenkinson M, Smith S (2001) A global optimisation method for robust affine registration of brain images. Med Image Anal 5: 143–156. 11516708 10.1016/s1361-8415(01)00036-6

[36] Dale AM, Fischl B, Sereno MI (1999) Cortical surface-based analysis. I. Segmentation and surface reconstruction. Neuroimage 9: 179–194. 9931268 10.1006/nimg.1998.0395

[37] Fischl B, Sereno MI, Dale AM (1999) Cortical surface-based analysis. II: Inflation, flattening, and a surface-based coordinate system. Neuroimage 9: 195–207. 9931269 10.1006/nimg.1998.0396

[38] Fischl B, Dale AM (2000) Measuring the thickness of the human cerebral cortex from magnetic resonance images. Proc Natl Acad Sci U S A 97: 11050–11055. 10984517 10.1073/pnas.200033797 PMC27146

[39] Salat DH, Buckner RL, Snyder AZ, Greve DN, Desikan RS, et al (2004) Thinning of the cerebral cortex in aging. Cereb Cortex 14: 721–730. 15054051 10.1093/cercor/bhh032

[40] Park HJ, Kim JJ, Lee SK, Seok JH, Chun J, et al (2008) Corpus callosal connection mapping using cortical gray matter parcellation and DT-MRI. Hum Brain Mapp 29: 503–516. 17133394 10.1002/hbm.20314 PMC6870924

[41] Sabatinelli D, Fortune EE, Li Q, Siddiqui A, Krafft C, et al (2011) Emotional perception: meta-analyses of face and natural scene processing. Neuroimage 54: 2524–2533. 20951215 10.1016/j.neuroimage.2010.10.011

[42] Haxby JV, Hoffman EA, Gobbini MI (2002) Human neural systems for face recognition and social communication. Biol Psychiatry 51: 59–67. 11801231 10.1016/s0006-3223(01)01330-0

[43] Atkinson AP, Adolphs R (2011) The neuropsychology of face perception: beyond simple dissociations and functional selectivity. Philos Trans R Soc Lond B Biol Sci 366: 1726–1738. 21536556 10.1098/rstb.2010.0349 PMC3130374

[44] Naikar N (1996) Perception of apparent motion of colored stimuli after commissurotomy. Neuropsychologia 34: 1041–1049. 8904741 10.1016/0028-3932(96)00019-x

[45] Symington SH, Paul LK, Symington MF, Ono M, Brown WS (2010) Social cognition in individuals with agenesis of the corpus callosum. Soc Neurosci 5: 296–308. 20162492 10.1080/17470910903462419

[46] Catani M, Jones DK, Donato R, Ffytche DH (2003) Occipito-temporal connections in the human brain. Brain 126: 2093–2107. 12821517 10.1093/brain/awg203

[47] Philippi CL, Mehta S, Grabowski T, Adolphs R, Rudrauf D (2009) Damage to association fiber tracts impairs recognition of the facial expression of emotion. J Neurosci 29: 15089–15099. 19955360 10.1523/JNEUROSCI.0796-09.2009 PMC2819193

[48] Wakana S, Jiang H, Nagae-Poetscher LM, van Zijl PC, Mori S (2004) Fiber tract-based atlas of human white matter anatomy. Radiology 230: 77–87. 14645885 10.1148/radiol.2301021640

[49] Duering M, Zieren N, Herve D, Jouvent E, Reyes S, et al (2011) Strategic role of frontal white matter tracts in vascular cognitive impairment: a voxel-based lesion-symptom mapping study in CADASIL. Brain 134: 2366–2375. 21764819 10.1093/brain/awr169

[50] Niogi S, Mukherjee P, Ghajar J, McCandliss BD (2010) Individual Differences in Distinct Components of Attention are Linked to Anatomical Variations in Distinct White Matter Tracts. Front Neuroanat 4: 2. 20204143 10.3389/neuro.05.002.2010 PMC2831631

[51] van Walderveen MA, Kamphorst W, Scheltens P, van Waesberghe JH, Ravid R, et al (1998) Histopathologic correlate of hypointense lesions on T1-weighted spin-echo MRI in multiple sclerosis. Neurology 50: 1282–1288. 9595975 10.1212/wnl.50.5.1282

[52] Summers M, Fisniku L, Anderson V, Miller D, Cipolotti L, et al (2008) Cognitive impairment in relapsing-remitting multiple sclerosis can be predicted by imaging performed several years earlier. Mult Scler 14: 197–204. 17986503 10.1177/1352458507082353

[53] Amato MP, Bartolozzi ML, Zipoli V, Portaccio E, Mortilla M, et al (2004) Neocortical volume decrease in relapsing-remitting MS patients with mild cognitive impairment. Neurology 63: 89–93. 15249616 10.1212/01.wnl.0000129544.79539.d5

[54] Sepulcre J, Goni J, Masdeu JC, Bejarano B, Velez de Mendizabal N, et al (2009) Contribution of white matter lesions to gray matter atrophy in multiple sclerosis: evidence from voxel-based analysis of T1 lesions in the visual pathway. Arch Neurol 66: 173–179. 19204153 10.1001/archneurol.2008.562

[55] Haxby JV, Hoffman EA, Gobbini MI (2000) The distributed human neural system for face perception. Trends Cogn Sci 4: 223–233. 10827445 10.1016/s1364-6613(00)01482-0

[56] Zaki J, Hennigan K, Weber J, Ochsner KN (2010) Social cognitive conflict resolution: contributions of domain-general and domain-specific neural systems. J Neurosci 30: 8481–8488. 20573895 10.1523/JNEUROSCI.0382-10.2010 PMC2916865

[57] Skelly LR, Decety J (2012) Passive and motivated perception of emotional faces: qualitative and quantitative changes in the face processing network. PLoS One 7: e40371. 22768287 10.1371/journal.pone.0040371 PMC3386961

[58] Bastiaansen JA, Thioux M, Keysers C (2009) Evidence for mirror systems in emotions. Philos Trans R Soc Lond B Biol Sci 364: 2391–2404. 19620110 10.1098/rstb.2009.0058 PMC2865077

[59] Carr L, Iacoboni M, Dubeau MC, Mazziotta JC, Lenzi GL (2003) Neural mechanisms of empathy in humans: a relay from neural systems for imitation to limbic areas. Proc Natl Acad Sci U S A 100: 5497–5502. 12682281 10.1073/pnas.0935845100 PMC154373

[60] Kuzmanovic B, Bente G, von Cramon DY, Schilbach L, Tittgemeyer M, et al (2012) Imaging first impressions: distinct neural processing of verbal and nonverbal social information. Neuroimage 60: 179–188. 22227133 10.1016/j.neuroimage.2011.12.046

[61] Leslie KR, Johnson-Frey SH, Grafton ST (2004) Functional imaging of face and hand imitation: towards a motor theory of empathy. Neuroimage 21: 601–607. 14980562 10.1016/j.neuroimage.2003.09.038

[62] Buccino G, Binkofski F, Fink GR, Fadiga L, Fogassi L, et al (2001) Action observation activates premotor and parietal areas in a somatotopic manner: an fMRI study. Eur J Neurosci 13: 400–404. 11168545

