# Supplementary material for: Disconnection Mechanism and Regional Cortical Atrophy Contribute to Impaired Processing of Facial Expressions and Theory of Mind in Multiple Sclerosis: A Structural MRI Study
Source: PLoS One. 2013 Dec 13;8(12):e82422. doi: 10.1371/journal.pone.0082422 (PMC3862626; doi:10.1371/journal.pone.0082422)
Supplement: Table S1 — Regional T1-lesion content of the white matter tracts from the ICBM DTI-81 Atlas, and correlations between mentalization test performance and regional T1-lesion loads in patients with multiple sclerosis. The volumes of each individual fiber bundle were assessed, and then their relative lesion content was calculated. Correlation between regional T1-lesion volumes and the social cognition test performances were analyzed with ANCOVA controlling for gender, EDSS, depression, and anxiety. P-value after Bonferroni correction was p<0.0024. Significant correlations are indicated with bold format. (DOCX) [file pone.0082422.s001.docx]

**Table S1.** Regional T1-lesion content of the white matter tracts from the ICBM DTI-81 Atlas, and correlations between mentalization test performance and regional T1-lesion loads in patients with multiple sclerosis.

| **Regional T1-lesion volumes** | | | | | | | | | | | |  |
| --- | --- | --- | --- | --- | --- | --- | --- | --- | --- | --- | --- | --- |
|  | | | Eyes test | | Faces test | | | | Faux pas test | | |  |
| White matter tracts | Relative lesion content (%): mean±StD (range) | N^[[1]](#footnote--1)^ | ^[[2]](#footnote-0)^ap | ^a^R^2^ | ^a^p | | ^a^R^2^ | | ^a^p | ^a^R^2^ | |  |
| Middle cerebellar peduncle (MCP) | 0.251±0.718 (0.000-4.422) | 21 | 0.367 | 0.155 | 0.469 | | 0.153 | | 0.615 | 0.217 | |  |
| Pontine crossing tract (a part of MCP) | 0.033±0.120 (0.000-0.733) | 5 | NA | | | | | | | | |  |
| **Genu of corpus callosum** | 0.881±1.457 (0.000-7.320) | 36 | 0.056 | 0.213 | | **<0.001** | | 0.415 | 0.040 | | 0.291 |  |
| Body of corpus callosum | 0.754±1.122 (0.000-4.962) | 42 | 0.017 | 0.253 | | 0.016 | | 0.259 | 0.078 | | 0.271 |  |
| **Splenium of corpus callosum** | 1.052±1.937 (0.000-6.968) | 35 | **0.002** | 0.317 | | **0.002** | | 0.332 | 0.012 | | 0.329 |  |
| Fornix (column and body of fornix) | 0.052±0.366 (0.000-2.564) | 1 | NA | | | | | | | | |  |
| Corticospinal tract right | 0.223±0.827 (0.000-4.981) | 6 |  |  |  |  |  |  |  |  |  |  |
| Corticospinal tract left | 0.334±1.583 (0.000-10.155) | 6 |  |  |  |  |  |  |  |  |  |  |
| Medial lemniscus right | 0.254±1.032 (0.000-6.274) | 6 |  |  |  |  |  |  |  |  |  |  |
| Medial lemniscus left | 0.471±1.537 (0.000-8.791) | 8 |  |  |  |  |  |  |  |  |  |  |
| Inferior cerebellar peduncle right | 0.064±0.282 (0.000-1.852) | 4 |  |  |  |  |  |  |  |  |  |  |
| Inferior cerebellar peduncle left | 0.444±1.565 (0.000-9.909) | 9 |  |  |  |  |  |  |  |  |  |  |
| Superior cerebellar peduncle right | 0.051±0.354 (0.000-2.476) | 1 |  |  |  |  |  |  |  |  |  |  |
| Superior cerebellar peduncle left | 0.143±0.644 (0.000-3.441) | 3 |  |  |  |  |  |  |  |  |  |  |
| Cerebral peduncle right | 0.142±0.790 (0.000-5.515) | 6 |  |  |  |  |  |  |  |  |  |  |
| Cerebral peduncle left | 0.056±0.260 (0.000-1.738) | 4 |  |  |  |  |  |  |  |  |  |  |
| Anterior limb of internal capsule right | 0.043±0.156 (0.000-0.772) | 7 |  |  |  |  |  |  |  |  |  |  |
| Anterior limb of internal capsule left | 0.148±0.794 (0.000-5.357) | 6 |  |  |  |  |  |  |  |  |  |  |
| Posterior limb of internal capsule right | 0.012±0.053 (0.000-0.281) | 3 |  |  |  |  |  |  |  |  |  |  |
| Posterior limb of internal capsule left | 0.076±0.230 (0.000-1.025) | 6 |  |  |  |  |  |  |  |  |  |  |
| Retrolenticular part of internal capsule right | 0.348±1.195 (0.000-7.854) | 13 | 0.158 | 0.179 | | 0.003 | | 0.315 | 0.142 | | 0.254 | |
| Retrolenticular part of internal capsule left | 0.771±2.076 (0.000-7.980) | 14 | 0.146 | 0.182 | | 0.003 | | 0.318 | 0.347 | | 0.229 | |
| Anterior corona radiata right | 1.442±3.033 (0.000-13.959) | 35 | 0.368 | 0.154 | | 0.120 | | 0.193 | 0.418 | | 0.225 | |
| **Anterior corona radiata left** | 1.116±2.006 (0.000-8.906) | 31 | 0.018 | 0.251 | | **<0.001** | | 0.416 | 0.030 | | 0.300 | |
| Superior corona radiata right | 1.005±1.752 (0.000-9.289) | 35 | 0.948 | 0.137 | | 0.396 | | 0.157 | 0.173 | | 0.248 | |
| Superior corona radiata left | 1.428±4.076 (0.000-27.384) | 34 | 0.129 | 0.186 | | 0.170 | | 0.182 | 0.103 | | 0.263 | |
| Posterior corona radiata right | 2.150±3.995 (0.000-18.715) | 29 | 0.017 | 0.252 | | 0.210 | | 0.175 | 0.030 | | 0.300 | |
| Posterior corona radiata left | 2.692±4.934 (0.000-26.654) | 33 | 0.117 | 0.189 | | 0.026 | | 0.243 | 0.062 | | 0.278 | |
| Posterior thalamic radiation (include optic radiation) right | 1.981±3.740 (0.000-18.801) | 34 | 0.644 | 0.142 | | 0.674 | | 0.145 | 0.827 | | 0.213 | |
| Posterior thalamic radiation (include optic radiation) left | 2.351±3.092 (0.000-11.845) | 36 | 0.163 | 0.178 | | 0.043 | | 0.226 | 0.040 | | 0.291 | |
| **Sagittal stratum (include inferior longitidinal fasciculus and inferior fronto-occipital fasciculus) right** | 0.804±2.229 (0.000-14.419) | 21 | 0.135 | 0.184 | | **<0.001** | | 0.407 | 0.333 | | 0.230 | |
| Sagittal stratum (include inferior longitidinal fasciculus and inferior fronto-occipital fasciculus) left | 2.052±3.969 (0.000-15.990) | 28 | 0.861 | 0.138 | | 0.609 | | 0.147 | 0.855 | | 0.212 | |
| External capsule right | 0.078±0.275 (0.000-1.789) | 10 | NA | | | | | | | | |  |
| External capsule left | 0.101±0.277 (0.000-1.596) | 11 |  |  |  |  |  |  |  |  |  |  |
| Cingulum (cingulate gyrus) right | 0.025±0.085 (0.000-0.350) | 4 |  |  |  |  |  |  |  |  |  |  |
| Cingulum (cingulate gyrus) left | 0.054±0.188 (0.000-0.993) | 5 |  |  |  |  |  |  |  |  |  |  |
| Cingulum (hippocampus) right | 0.062±0.276 (0.000-1.442) | 3 |  |  |  |  |  |  |  |  |  |  |
| Cingulum (hippocampus) left | 0.097±0.597 (0.000-4.173) | 3 |  |  |  |  |  |  |  |  |  |  |
| Fornix (cres) / Stria terminalis right | 0.035±0.145 (0.000-0.980) | 6 |  |  |  |  |  |  |  |  |  |  |
| Fornix (cres) / Stria terminalis left | 0.118±0.668 (0.000-4.670) | 6 |  |  |  |  |  |  |  |  |  |  |
| Superior longitudinal fasciculus right | 0.288±0.699 (0.000-3.115) | 24 | 0.562 | 0.144 | | 0.766 | 0.144 | | 0.320 | | 0.231 | |
| Superior longitudinal fasciculus left | 0.416±1.054 (0.000-6.204) | 24 | 0.215 | 0.170 | | 0.285 | 0.166 | | 0.432 | | 0.224 | |
| Superior fronto-occipital fasciculus (could be a part of anterior internal capsule) right | 0.518±1.838 (0.000-9.892) | 8 | NA | | | | | | | | |  |
| Superior fronto-occipital fasciculus (could be a part of anterior internal capsule) left | 1.112±4.691 (0.000-32.083) | 14 | 0.098 | 0.195 | | 0.036 | 0.232 | | 0.047 | | 0.287 | |
| Inferior fronto-occipital fasciculus right | 0.000±0.000 (0.000-0.000) | 0 | NA | | | | | | | | |  |
| Inferior fronto-occipital fasciculus left | 0.105±0.458 (0.000-2.871) | 4 |  |  |  |  |  |  |  |  |  |  |
| Uncinate fasciculus right | 6.667±11.253 (0.000-43.951) | 32 | 0.051 | 0.216 | | **0.002** | | 0.325 | 0.021 | | 0.312 | |
| Uncinate fasciculus left | 6.691±11.699 (0.000-52.021) | 31 | 0.022 | 0.244 | | **<0.001** | | 0.468 | 0.011 | | 0.331 | |

The volumes of each individual fiber bundle were assessed, and then their relative lesion content was calculated. Correlation between regional T1-lesion volumes and the social cognition test performances were analyzed with ANCOVA controlling for gender, EDSS, depression, and anxiety. P-value after Bonferroni correction was p<0.0024. Significant correlations are indicated with bold format.

1. number of patients with lesions in the given fiber tract [↑](#footnote-ref--1)
2. a correlations were only examined if ≥25% (N≥12) of the patients were affected by lesions in the given fiber tract [↑](#footnote-ref-0)
