# Supplementary material for: Disconnection Mechanism and Regional Cortical Atrophy Contribute to Impaired Processing of Facial Expressions and Theory of Mind in Multiple Sclerosis: A Structural MRI Study
Source: PLoS One. 2013 Dec 13;8(12):e82422. doi: 10.1371/journal.pone.0082422 (PMC3862626; doi:10.1371/journal.pone.0082422)
Supplement: Table S2 — Regional T2-lesion content of the white matter tracts from the ICBM DTI-81 Atlas, and correlations between mentalization test performance and regional T2-lesion loads in patients with multiple sclerosis. The volumes of each individual fiber bundle were assessed, and then their relative lesion content was calculated. Correlation between regional T2-lesion volumes and social cognition test performances were analyzed with ANCOVA controlling for gender, EDSS, depression, and anxiety. P-value after Bonferroni correction was p<0.0014. Significant correlations are indicated with bold format. (DOCX) [file pone.0082422.s002.docx]

**Table S2.** Regional T2-lesion content of the white matter tracts from the ICBM DTI-81 Atlas, and correlations between mentalization test performance and regional T2-lesion loads in patients with multiple sclerosis.

| **Regional T2-lesion volumes** | | | | | | | | |
| --- | --- | --- | --- | --- | --- | --- | --- | --- |
|  | | | Eyes test | | Faces test | | Faux pas test | |
| White matter tracts | Relative lesion content (%): mean±StD (range) | N^[[1]](#footnote--1)^ | ^[[2]](#footnote-0)^ap | ^a^R^2^ | ^a^p | ^a^R^2^ | ^a^p | ^a^R^2^ |
| Middle cerebellar peduncle (MCP) | 0.51216±1.446 (0.0000-9.111) | 24 | 0.129 | 0.186 | 0.728 | 0.144 | 0.347 | 0.229 |
| Pontine crossing tract (a part of MCP) | 0.60710±3.551 (0.000-24.812) | 6 | NA | | | | | |
| **Genu of corpus callosum** | 7.74794±6.439 (0.251-27.723) | 49 | 0.081 | 0.201 | **<0.001** | 0.369 | 0.124 | 0.258 |
| Body of corpus callosum | 9.48737±7.831 (0.000-27.240) | 48 | 0.139 | 0.183 | 0.034 | 0.234 | 0.210 | 0.242 |
| Splenium of corpus callosum | 13.76224±11.360 (0.000-33.027) | 48 | 0.016 | 0.255 | 0.013 | 0.266 | 0.150 | 0.252 |
| Fornix (column and body of fornix) | 5.90827±5.606 (0.000-24.425) | 43 | 0.384 | 0.153 | 0.897 | 0.142 | 0.079 | 0.271 |
| Corticospinal tract right | 0.32673±1.116 (0.000-5.824) | 7 | NA | | | | | |
| Corticospinal tract left | 0.98259±3.436 (0.000-17.421) | 8 |  |  |  |  |  |  |
| Medial lemniscus right | 1.60653±7.846 (0.000-53.418) | 5 |  |  |  |  |  |  |
| Medial lemniscus left | 2.13692±9.656 (0.000-66.239) | 11 |  |  |  |  |  |  |
| Inferior cerebellar peduncle right | 0.46327±2.255 (0.000-14.763) | 6 |  |  |  |  |  |  |
| Inferior cerebellar peduncle left | 2.58129±4.729 (0.000-16.830) | 18 | 0.510 | 0.146 | 0.058 | 0.216 | 0.887 | 0.212 |
| Superior cerebellar peduncle right | 0.62994±2.312 (0.000-12.210) | 6 | NA | | | | | |
| Superior cerebellar peduncle left | 1.79422±4.811 (0.000-23.618) | 14 | 0.118 | 0.189 | 0.437 | 0.155 | 0.984 | 0.212 |
| Cerebral peduncle right | 0.29488±1.444 (0.000-9.477) | 4 | NA | | | | | |
| Cerebral peduncle left | 0.23608±0.827 (0.000-4.472) | 5 |  |  |  |  |  |  |
| Anterior limb of internal capsule right | 0.95036±2.279 (0.000-13.413) | 23 | 0.504 | 0.147 | 0.101 | 0.198 | 0.087 | 0.268 |
| Anterior limb of internal capsule left | 1.69184±6.636 (0.000-45.709) | 22 | 0.184 | 0.175 | 0.112 | 0.195 | 0.065 | 0.277 |
| Posterior limb of internal capsule right | 0.86055±1.802 (0.000-8.848) | 22 | 0.277 | 0.162 | 0.930 | 0.142 | 0.603 | 0.217 |
| Posterior limb of internal capsule left | 2.59667±6.398 (0.000-36.313) | 23 | 0.587 | 0.143 | 0.197 | 0.177 | 0.119 | 0.259 |
| Retrolenticular part of internal capsule right | 8.38271±11.877 (0.000-40.885) | 33 | 0.284 | 0.162 | 0.138 | 0.188 | 0.066 | 0.276 |
| Retrolenticular part of internal capsule left | 5.59739±9.362 (0.000-43.096) | 30 | 0.229 | 0.198 | 0.017 | 0.257 | 0.107 | 0.262 |
| Anterior corona radiata right | 8.12022±9.659 (0.000-43.560) | 48 | 0.097 | 0.195 | 0.003 | 0.316 | 0.149 | 0.252 |
| Anterior corona radiata left | 8.00708±10.527 (0.000-44.024) | 48 | 0.130 | 0.186 | 0.002 | 0.319 | 0.064 | 0.277 |
| Superior corona radiata right | 15.40259±16.012 (0.192-65.126) | 49 | 0.096 | 0.195 | 0.124 | 0.191 | 0.195 | 0.244 |
| Superior corona radiata left | 15.07947±18.864 (0.000-90.357) | 46 | 0.186 | 0.174 | 0.084 | 0.204 | 0.216 | 0.242 |
| Posterior corona radiata right | 35.21365±25.747 (0.043-81.598) | 49 | 0.022 | 0.244 | 0.143 | 0.187 | 0.187 | 0.246 |
| Posterior corona radiata left | 33.03800±25.514 (0.000-84.712) | 48 | 0.114 | 0.190 | 0.139 | 0.188 | 0.480 | 0.222 |
| Posterior thalamic radiation (include optic radiation) right | 30.43418±22.450 (0.000-74.988) | 48 | 0.372 | 0.372 | 0.395 | 0.157 | 0.514 | 0.220 |
| Posterior thalamic radiation (include optic radiation) left | 27.06204±18.625 (0.783-62.802) | 49 | 0.519 | 0.146 | 0.233 | 0.172 | 0.626 | 0.216 |
| Sagittal stratum (include inferior longitidinal fasciculus and inferior fronto-occipital fasciculus) right | 20.54867±23.647 (0.000-71.414) | 39 | 0.379 | 0.154 | 0.059 | 0.216 | 0.856 | 0.212 |
| Sagittal stratum (include inferior longitidinal fasciculus and inferior fronto-occipital fasciculus) left | 14.93318±17.378 (0.000-63.083) | 38 | 0.499 | 0.147 | 0.055 | 0.218 | 0.356 | 0.229 |
| External capsule right | 0.75273±1.432 (0.000-5.911) | 22 | 0.285 | 0.162 | 0.748 | 0.144 | 0.729 | 0.214 |
| External capsule left | 0.86235±1.491 (0.000-6.352) | 20 | 0.713 | 0.140 | 0.440 | 0.154 | 0.042 | 0.290 |
| Cingulum (cingulate gyrus) right | 0.26731±0.652 (0.000-3.215) | 16 | 0.179 | 0.175 | 0.273 | 0.167 | 0.907 | 0.212 |
| Cingulum (cingulate gyrus) left | 0.54586±1.177 (0.000-4.948) | 17 | 0.011 | 0.268 | 0.011 | 0.272 | 0.162 | 0.250 |
| Cingulum (hippocampus) right | 1.83733±4.965 (0.000-28.270) | 18 | 0.641 | 0.142 | 0.382 | 0.158 | 0.986 | 0.212 |
| Cingulum (hippocampus) left | 1.40861±4.322 (0.000-27.431) | 16 | 0.113 | 0.190 | 0.608 | 0.147 | 0.798 | 0.213 |
| Fornix (cres) / Stria terminalis right | 3.38157±6.493 (0.000-28.511) | 24 | 0.113 | 0.190 | 0.019 | 0.253 | 0.130 | 0.256 |
| **Fornix (cres) / Stria terminalis left** | 2.58451±6.032 (0.000-30.000) | 17 | 0.005 | 0.296 | **<0.001** | 0.471 | 0.067 | 0.276 |
| Superior longitudinal fasciculus right | 5.34271±6.655 (0.000-23.901) | 40 | 0.016 | 0.255 | 0.409 | 0.156 | 0.731 | 0.214 |
| Superior longitudinal fasciculus left | 3.37576±7.011 (0.000-37.171) | 39 | 0.317 | 0.158 | 0.330 | 0.162 | 0.454 | 0.223 |
| Superior fronto-occipital fasciculus (could be a part of anterior internal capsule) right | 10.14159±16.928 (0.000-86.260) | 33 | 0.188 | 0.174 | 0.015 | 0.262 | 0.073 | 0.273 |
| Superior fronto-occipital fasciculus (could be a part of anterior internal capsule) left | 9.96935±17.472 (0.000-100.000) | 31 | 0.097 | 0.195 | 0.078 | 0.206 | 0.098 | 0.264 |
| Inferior fronto-occipital fasciculus right | 0.62182±3.484 (0.000-24.224) | 4 | NA | | | | | |
| Inferior fronto-occipital fasciculus left | 0.03692±0.181 (0.000-0.935) | 2 |  |  |  |  |  |  |
| Uncinate fasciculus right | 61.56633±29.208 (0.000-100.000) | 48 | 0.096 | 0.195 | 0.366 | 0.159 | 0.294 | 0.233 |
| Uncinate fasciculus left | 57.08290±29.146 (0.000-99.191) | 47 | 0.027 | 0.237 | 0.076 | 0.207 | 0.305 | 0.232 |

The volumes of each individual fiber bundle were assessed, and then their relative lesion content was calculated. Correlation between regional T2-lesion volumes and social cognition test performances were analyzed with ANCOVA controlling for gender, EDSS, depression, and anxiety. P-value after Bonferroni correction was p<0.0014. Significant correlations are indicated with bold format.

1. number of patients with lesions in the given fiber tract [↑](#footnote-ref--1)
2. a correlations were only examined if ≥25% (N≥12) of the patients were affected by lesions in the given fiber tract [↑](#footnote-ref-0)
