# Supplementary material for: Disconnection Mechanism and Regional Cortical Atrophy Contribute to Impaired Processing of Facial Expressions and Theory of Mind in Multiple Sclerosis: A Structural MRI Study
Source: PLoS One. 2013 Dec 13;8(12):e82422. doi: 10.1371/journal.pone.0082422 (PMC3862626; doi:10.1371/journal.pone.0082422)
Supplement: Text S1 — Example from the Faux pas test. The presented story contains a faux pas situation. (DOCX) [file pone.0082422.s003.docx]

**Text S1**

**Example for a faux pas story**

Jill had just moved into a new apartment. Jill went shopping and bought some new curtains for her bedroom. When she had just finished decorating the apartment, her best friend, Lisa, came over. Jill gave her a tour of the apartment and asked, "How do you like my bedroom?" "Those curtains are horrible," Lisa said. "I hope you're going to get some new ones!"

Questions:

1. Did Lisa know the curtains were new?
2. Did someone say something he shouldn’t have said?
